# CRANIAL OSTEOMYELITIS AS A COMPLICATION OF FURUNCULAR MYIASIS

**DOI:** 10.1590/1984-0462/2021/39/2020105

**Published:** 2021-02-05

**Authors:** Nelson Muñoz, Sandra Galvis, Oscar Patiño, Carlos Moneriz

**Affiliations:** a Universidad de Cartagena, Cartagena, Colombia.; b Napoleon Franco Pareja Children’s Hospital, “Children’s House”, Cartagena, Colombia.

**Keywords:** Myiasis, Osteomyelitis, Larvae, Hypodermyiasis, Miíase, Osteomielite, Larva, Hipodermose

## Abstract

**Objective:**

To report the case of an infant with infrequent cranial osteomyelitis as a complication of furuncular myiasis.

**Case description:**

The patient was a 4-month-old male who presented to the emergency department with a nodular skull lesion with edema, tenderness, pain, and purulent drainage, as well as progress of the ulcerated lesion and evidence of larvae inside. Antibiotic treatment was initiated, and the patient was taken to the operating room to remove the larvae, but he had no symptomatic improvement. A skull radiograph was taken to visualize the osteolytic lesion, and a 3D computed tomography scan showed osteomyelitis of the external parietal surface. Antibiotic management readjustment continued for a total of six weeks, and a skin flap was used with clinical improvement.

**Comments:**

Myiasis is defined as the infestation of vertebrates with fly larvae. In mammals, larvae can feed on host tissue and cause a wide range of infestations depending on their location in the body. The cranial osteomyelitis as a complication of myiasis described in this report seems to be an exceptional case.

## RESUMO

**Objetivo:**

Relatar um caso de criança com osteomielite craniana infrequente como complicação da miíase furuncular.

**Descrição do caso:**

Paciente do sexo masculino, com quatro meses de idade, que se apresentou no pronto-socorro com lesão nodular no crânio com edema, sensibilidade, dor e drenagem purulenta, com evolução da lesão ulcerada e evidência de larva no interior. O tratamento com antibióticos foi iniciado e o paciente foi levado à sala de cirurgia para remover as larvas, mas não houve melhora. Uma radiografia do crânio foi realizada para visualizar a lesão osteolítica e uma tomografia computadorizada em 3D mostrou osteomielite da superfície parietal externa. O reajuste do tratamento com antibióticos foi mantido por um total de seis semanas e um retalho cutâneo foi realizado com melhora clínica.

**Comentários:**

Miíase é definida como a infestação de vertebrados com larvas de moscas. Nos mamíferos, as larvas podem se alimentar do tecido hospedeiro e causar uma ampla variedade de infestações, dependendo da sua localização no corpo. A osteomielite como complicação da miíase, apresentada nesse caso, parece ser uma forma não usual de complicação dessa doença.

## INTRODUCTION

Myiasis is defined as the infestation of a vertebrate host by fly larvae that feed on living tissue or body fluids.^1^ ^,^ ^2^ The infection develops as a result of the maturation of eggs deposited on intact or damaged skin, ulcers of the lower limbs, various wounds, and tumors.^3^ The main species of flies that cause these infections are *Dermatobia hominis* (human botfly)^4^ from Central and Latin America and *Cordylobia anthropophaga* (tumbu fly)^5^ ^,^ ^6^ from Africa. Myiasis can also be acquired by travelers or tourists from other countries visiting these areas.^2^ ^,^ ^6^ ^,^ ^7^

*D. hominis* is native to Central and South America. Their larvae are transmitted to vertebrate animals by blood-sucking insects.^8^ When the blood-feeding vector finds a warm-blooded animal, the change in temperature causes the eggs of the flies to hatch.^9^ Larvae enter the vertebrate host through a hair follicle, the bite site, or by directly burrowing into the skin. Over the next four to 18 weeks, the larva grows by eating the host’s tissue.^2^ At maturity, the larva emerges from the wound and falls to the ground until reaching the pupa stage (last stage of the larva). Despite its name, *D. hominis* also infests cattle, monkeys, rodents, and birds.^2^ ^,^ ^10^

The most frequent clinical manifestations of myiasis are itching, pain, and sensation of movement, and they generally occur suddenly at night before fluid drainage.^11^ Furuncular injury is the most common presentation of this pathology and almost always heals completely, without a trace. The most commonly reported complication of furuncular lesions is bacterial infection.^3^ In rare cases, osteomyelitis can occur, but the information found in the literature^12^ was scarce. The present work reports an unusual case of cranial osteomyelitis as a complication of furuncular myiasis.

## CASE DESCRIPTION

**Figure 1:**
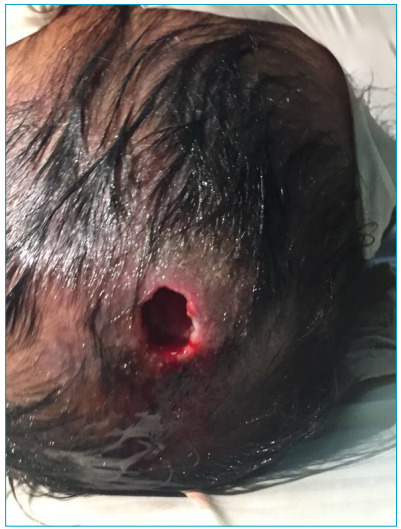
Cranial osteomyelitis as a complication of furuncular myiasis in an infant. The image shows an ulcer of 3 cm in diameter with purulent drainage, peristomal erythema, edema, tenderness, and pain.

The patient was a 4-month-old male, from a low-income area in the city of Cartagena de Indias (Colombia), with a history of seborrheic dermatitis and tinea capitis. He presented to the emergency room with a one-week fever, associated with a nodular skull lesion in the parietal region with edema, tenderness, pain, and spontaneous purulent drainage. Oral antibiotic therapy was started without improvement. The lesion progressed to an ulcer of approximately 3 cm in diameter (Figure 1), and upon admission to the hospital, *D. hominis* larvae were evident through the ulcerated orifice.

**Figure 2:**
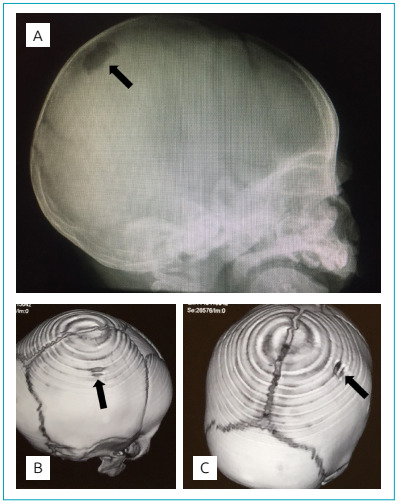
X-ray (A) and 3D computed tomography images (B and C) of the skull of the infant with myiasis. The images show lytic lesions in the parietal bone, confirming the osteomyelitis of the external surface of the skull bone.

Treatment with cephalothin was initiated, and a surgical procedure was performed to remove the larvae, finding approximately ten larvae. However, the patient persisted with fever, purulent drainage, and presence of larvae even after 6 days of antibiotic treatment; thus, clindamycin and ivermectin administration was started. Skull radiograph showed an osteolytic lesion, and 3D-reconstructed computed tomography confirmed osteomyelitis of the external surface of the parietal bone (Figure 2). The patient was treated for a total of six weeks with clindamycin and four weeks with rifampicin, and a scalp flap was also used. Study for primary and secondary human immunodeficiency virus (HIV) was negative. The patient was discharged, continuing outpatient antibiotic management, and follow-up appointments.

## DISCUSSION

Cutaneous myiasis is a prevalent disease in tropical or subtropical countries from Central America, South America, and Africa. The fly species *D. hominis* is the most commonly involved.^4^ Lack of hygiene is the main factor for the incidence of the disease.^1^ This patient was from a family of low economic resources and poor hygienic conditions. In addition, he lived in Cartagena, a city located in Colombia characterized by a very humid and tropical climate. Another important risk factor in this patient was the abundance of exposed pre-existing suppurative lesions that attracted and stimulated egg deposition by the female insect.^3^ ^,^ ^11^

**Figure 3:**
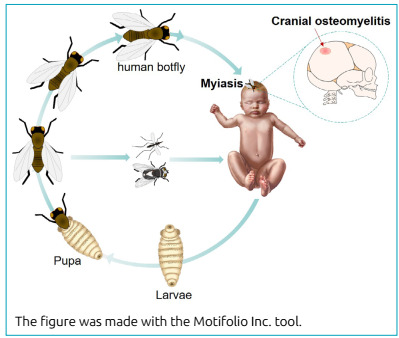
Stages of human myiasis. The female human botfly lays eggs directly in human tissue or the belly of other flies and various mosquitoes. When the fly or vector lands on a vertebrate, the eggs hatch from the heat. The larvae then grow under the skin, entering through a hair follicle, wound, or the bite site. In this reported patient, the furuncular myiasis of the scalp caused infrequent cranial osteomyelitis.

In this infant, myiasis originated from the infestation of *D. hominis* larvae, which enter the skin through the hair follicle, the bite site, or directly through an opening in the skin, as occurred in this case (Figure 3). Over the next few weeks, the larvae grow by feeding on the tissue of their host,^9^ producing lesions that are often misdiagnosed as a furuncle and progressing to a cavity, with an external opening that the larvae use to breathe and eliminate their excretions. Due to the natural cycle of the larvae, taking into account the antecedents of the insect bite is important to make a diagnosis.^11^

Cutaneous myiasis can be subdivided into three categories: localized furuncular myiasis, migratory myiasis, and wound myiasis, which occurs when fly larvae infest open wounds of the host. Furuncular presentation, corresponding to this case, is characterized by an itchy and erythematous papule in the bite area, later transforming into a furuncle with central depression.^3^ ^,^ ^11^ ^,^ ^13^ The diagnosis is basically clinical, taking into consideration the history of exposure, although the use of ultrasonography and dermoscopy is also recommended in some cases to confirm the diagnosis.^14^

The presumptive mechanism by which myiasis can develop into osteomyelitis is explained by the same pathophysiology of osteomyelitis, since one of the mechanisms for the development of bone infection is through a hematogenous route, with one of the predisposing factors being skin infection.^15^ The other possible route results from the local spread of a contaminated source adjacent to the bone lesion, but this scenario is more frequently associated with trauma, bone surgery, or hip replacement, a history that was not present in this case.^15^ However, no studies have been found that specifically describe the mechanism by which myiasis triggers osteomyelitis, so these statements are inconclusive.

In children, itching, distress, and difficulty maintaining sleep are common symptoms.^11^ If the infection occurs around skull holes, the risk of complications increases because the larvae can migrate into the nasal cavity, eyes, or brain tissue, and potentially cause blindness, sepsis, and death.^11^ The most important complications of myiasis are bacterial superinfection of the wound and tetanus. Nonetheless, secondary infections have been reported as rare due to bacteriostatic substances in the larva’s intestine.^12^

Myiasis has different treatment options; among them, the use of oral ivermectin is common, particularly for ocular and oral effects. Occlusion of the hole with Vaseline can lead to hypoxia in larvae and eggs, causing them to emerge through the central hole, or the larvae can be extracted with lateral pressure. The ideal treatment is the surgical removal of the larvae. However, cases in which parts of the body of the larvae are left in the wound must be taken into account, since this could trigger reactions by a foreign body.^13^

Despite the fact that myiasis morbidity is generally minimal, the association of this pathology with a superinfected wound increased the probability of the development of bacterial osteomyelitis in the skull of this patient. Therefore, surgical removal of the larvae, prolonged use of antibiotics, additional imaging studies, and a skin flap were required to avoid a greater possibility of complications or fatal outcomes, given the development of osteomyelitis. The complication described in this case of myiasis is rare and has an exceptional course, adding to the lack of other reports with similar developments.

The present report led us to conclude that myiasis generally occurs in a self-limited form, with easy resolution and low morbidity. Nevertheless, in this report, we discussed the course of a case of myiasis with an unusual complication of cranial osteomyelitis, warranting a longer hospital stay, longer antibiotic therapy, and higher risk of mortality. The report of this exceptional case will allow the expansion of knowledge to understand the disease and its possible complications.

## References

[1] Özkol HU, Calka O (2014). Furuncle persistent to long-term antibiotic therapy in a non-tropical region: a diagnosis that must not be overlooked: furuncular cutaneous myiasis. Turkiye Parazitol Derg.

[2] Bhandari R, Janos DP, Sinnis P (2007). Furuncular myiasis caused by Dermatobia hominis in a returning traveler. Am J Trop Med Hyg.

[3] McGraw TA, Turiansky GW (2008). Cutaneous myiasis. J Am Acad Dermatol.

[4] Boggild AK, Keystone JS, Kain KC (2002). Furuncular myiasis: a simple and rapid method for extraction of intact Dermatobia homini. Clin Infect Dis.

[5] Nawas ZY, Tong Y, Kollipara R, Peranteau AJ, Woc-Colburn L, Yan AC (2016). Emerging infectious diseases with cutaneous manifestations: Viral and bacterial infections. J Am Acad Dermatol.

[6] Song SM, Kim SW, Goo YK, Hong Y, Ock M, Cha HJ (2017). A case of furuncular myiasis due to Cordylobia anthropophaga in a Korean traveler returning from Uganda. Korean J Parasitol.

[7] Oliva E, Bargiggia G, Quinzan G, Lanza P, Farina C (2020). Furuncular myiasis in Italian traveler returning from Kenya. J Infect Dev Ctries.

[8] Shenouda M, Enten G, Nguyen T, Mangar D, Camporesi E (2018). Human botfly: a case report and overview of differential diagnosis. J Investig Med High Impact Case Rep.

[9] Maier H, Hönigsmann H. (2004). Furuncular myiasis caused by Dermatobia hominis, the human botfly. J Am Acad Dermatol.

[10] Hohenstein EJ, Buechner SA (2004). Cutaneous myiasis due to Dermatobia hominis. Dermatology.

[11] Francesconi F, Lupi O (2012). Myiasis. Clin Microbiol Rev.

[12] Vijay K, Kalapos P, Makkar A, Engbrecht B, Agarwal A (2013). Human botfly (Dermatobia hominis) larva in a child’s scalp mimicking osteomyelitis. Emerg Radiol.

[13] Solomon M, Lachish T, Schwartz E (2016). Cutaneous myiasis. Curr Infect Dis Rep.

[14] Graveriau C, Peyron F (2017). Cutaneous myiasis. Travel Med Infect Dis.

[15] Lew DP, Waldvogel FA (2004). Osteomyelitis. Lancet.

